# A61 GLOBAL INCIDENCE OF APPENDICITIS: A POPULATION-BASED STUDY OF THE ORGANISATION FOR ECONOMIC CO-OPERATION AND DEVELOPMENT

**DOI:** 10.1093/jcag/gwae059.061

**Published:** 2025-02-10

**Authors:** E Buie, S Coward, M J Buie, J A King, L M Wilson, M Quan, T Gimon, G G Kaplan

**Affiliations:** Community Health, University of Calgary, Calgary, AB, Canada; University of Calgary, Calgary, AB, Canada; University of Calgary, Calgary, AB, Canada; Community Health Sciences, University of Calgary, Calgary, AB, Canada; Community Health, University of Calgary, Calgary, AB, Canada; Community Health, University of Calgary, Calgary, AB, Canada; Community Health, University of Calgary, Calgary, AB, Canada; Community Health, University of Calgary, Calgary, AB, Canada

## Abstract

**Background:**

The global incidence of appendicitis in 2019 was estimated at 228 per 100,000 person-years. However, temporal trends of appendicitis rates vary between early industrialized and newly industrialized regions.

**Aims:**

To analyze annual appendectomy rates in regions of the Organisation for Economic Co-operation and Development (OECD) in the 21^st^ century.

**Methods:**

We conducted an observational population-based cohort study using data from 34 OECD regions from 2000–2023. OECD data provides country-level annual hospitalization rates for appendectomy per 100,000 person-years. We used Poisson regression to calculate Average Annual Percentage Change (AAPC) in appendectomy rates, with associated 95% confidence intervals (CI) for each region. CIs crossing 0% were defined as stable.

**Results:**

We observed geographic variation in appendectomy incidence rates, with rates ranging from 56.8 per 100,000 in Portugal (2023) to 165.6 per 100,000 in Switzerland (2022) (Table 1). Appendectomy rates significantly decreased in 22 regions and significantly increased in 10 regions, with AAPCs ranging from −4.25% (95%CI: −4.28, −4.22) in Italy to 1.46% (95%CI: 1.23, 1.68) in Norway (Table 1). AAPCs in Iceland were stable, and Latvia had insufficient data for time trend analysis.

**Conclusions:**

In the 21^st^ century, time trends of appendectomy rates across OECD regions displayed variation, with the majority decreasing. Geographic variability in rates and trends over time may be due to factors such as differential access to improved diagnostic imaging and non-surgical treatments.

Table 1. The Average Annual Percentage Change (AAPC) in appendectomy for the 34 regions of the OECD dataset with the corresponding year ranges for each region, confidence intervals, and associated average incidence per 100,000 person-years. All region AAPCs are significantly increasing or decreasing except Iceland and Latvia.

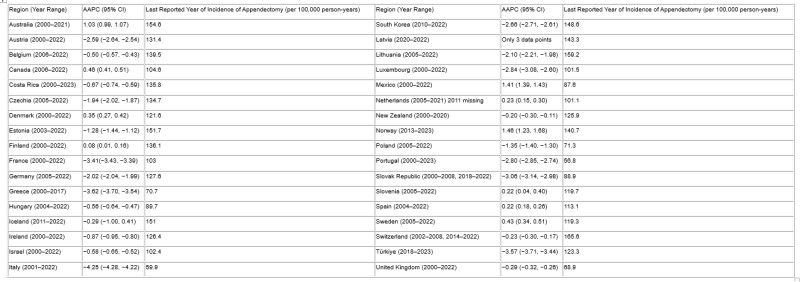

**Funding Agencies:**

None

